# V-doped MoS_2_ nanozymes providing reactive oxygen species and depleting glutathione for photothermally-enhanced nanocatalytic therapy

**DOI:** 10.3389/fphar.2024.1448867

**Published:** 2024-07-19

**Authors:** Haiyan Wang, Pengle Xia, Mekhrdod S. Kurboniyon, Shuhong Fang, Kunying Huang, Shufang Ning, Guanqiao Jin, Litu Zhang, Chen Wang

**Affiliations:** ^1^ Department of Research and Guangxi Cancer Molecular Medicine Engineering Research Center and Guangxi Key Laboratory of Basic and Translational Research for Colorectal Cancer, Guangxi Medical University Cancer Hospital, Nanning, China; ^2^ National Academy of Sciences of Tajikistan, Dushanbe, Tajikistan

**Keywords:** nanozymes, reactive oxygen species, glutathione, photothermal effect, tumor therapy

## Abstract

**Introduction:** The tumor microenvironment and multidrug resistance of tumor cells seriously impair the activity of the nanozymes.

**Methods:** Herein, a polyethylene glycol (PEG)-modified vanadium-doped molybdenum disulfide (V-MoS_2_@PEG) nanozymes were constructed to enhance anti-tumor activity through multi-enzymatic catalysis and photothermal effect with simultaneous reactive oxygen species replenishment and glutathione depletion.

**Results and discussion:** V-MoS_2_@PEG nanosheets exerted peroxidase activity by causing molybdenum ion (Mo^4+^) to react with hydrogen peroxide to form toxic hydroxyl radicals (·OH). Meanwhile, the V-doping can deplete glutathione avoiding ·OH consumption. In addition, the high heat generated by V-MoS_2_@PEG nanozymes under near-infrared laser irradiation brought about a desirable local temperature gradient, which produced an enhanced catalytic effect by promoting band bending. Furthermore, the photothermally inspired polarized charge increased the permeability of the tumor cell membrane and promoted further aggregation of the nanozymes, which realized the combination of photothermal therapy with multi-enzymatic catalysis, solved the problem of multi-enzyme catalysis, and improved the anti-tumor efficiency.

## 1 Introduction

With increasing global morbidity and mortality, cancer still poses a serious threat to human life and health. Since conventional treatment modalities (e.g., chemotherapy, radiotherapy, and surgical resection) have difficulty in achieving specific effects on malignant cells, these treatment modalities inevitably cause irreversible damage to normal cells and tissues in the body, resulting in poor patient prognosis. With the cross-vergence of nanomedicine and nanocatalysis, the utilization of nanozymes to trigger enzyme-catalyzed reactions under the unique physiological characteristics of the tumor microenvironment to achieve catalytic therapy with substrate specificity and low toxic side effects has become a novel strategy for tumor treatment. However, due to the complexity of the tumor microenvironment, the catalytic activity of nanozymes is often compromised under physiological conditions to cause incomplete tumor growth inhibition, which motivates researchers to amplify the tumor inhibition effect by modulating the tumor microenvironment and developing multi-modal synergistic therapies. Tumor microenvironment (TME) refers to the microenvironment of tumor cells and their surroundings, which is closely related to the formation and metastasis of tumors (Li et al., 2018; Mishra et al., 2021; Sindhu et al., 2021). TME includes not only peripheral microvessels, fibroblasts, lymphocytes, immune cells, bone marrow-derived inflammatory cell signaling molecules, and other extracellular matrices but also a variety of biological features within tumor tissues, such as oxygen content, pH, and redox environment (Dong et al., 2021). The use of abnormal biochemical markers in the tumor tissue microenvironment and tumor cells is a potential target for exploring and developing new therapeutic modalities.

Reactive oxygen species (ROS) are a class of highly oxidative molecules that are by-products of aerobic metabolism and a specific cell signaling transcription factor. ROS in living organisms mainly includes superoxide radicals (·O^2−^), hydrogen peroxide (H_2_O_2_), singlet oxygen (^1^O_2_), and hydroxyl radicals (·OH), etc. (Asgharzadeh et al., 2017; Zhang et al., 2017; Sawant and Lilly, 2020; Wu et al., 2022). ROS is not only implicated in protein folding, cell proliferation, differentiation and migration, and signaling but also in respiration, defensive responses, and oxidative damage. In biological cells, H_2_O_2_ is one of the most common ROS, mainly derived from the single-electron reduction of oxygen molecules, first to form O^2−^, and then catalyzed by superoxide dismutase to form H_2_O_2_ (Willems et al., 2015; Ferreira et al., 2018; Yang et al., 2018; Zhang C. et al., 2020; Yang and Lian, 2020; Sahu et al., 2022; Zhang et al., 2022). H_2_O_2_ has a broad range of catalytic activities and has been extensively used as a feedstock for activating drug release and generating intracellular oxygen. Tumor cells produce higher concentrations of H_2_O_2_ compared to normal cells. H_2_O_2_ can induce DNA damage and mutations, and increased H_2_O_2_ concentrations also activate hypoxia-inducible factor-1 (Masoud and Li, 2015; Ma et al., 2022). Activated HIF-1 plays an essential role in apoptosis, invasion, metastasis, and angiogenesis (Albadari et al., 2019; Infantino et al., 2021). H_2_O_2_ is highly biotoxic because it can be converted into various reactive free radicals, which can disrupt the redox metabolic balance in cells, especially by interacting with transition metal ions to generate highly reactive ·OH.

In addition, the regulation of the nanozymes can also affect their temperature to increase the catalytic activity of the nano-enzymes, leading to better and more effective tumor catalysis. Photothermal therapy (PTT), which collaboratively enhances the catalytic activity and therapeutic efficacy of nanozymes through the photothermal effect, is regarded as a promising therapeutic strategy (Wang et al., 2019; Zhang Q. et al., 2020; Yang et al., 2020; Zhou et al., 2020). For example, Fan and colleagues developed yolk-shell gold@carbon nanozymes, in which a synergistic effect of photothermal enhancement of enzyme-like activity was observed, exhibiting efficient tumor-destroying capabilities (Fan et al., 2018). Additionally, a MnO_2_@HMCu_2–x_S nanocomposite with photothermally enhanced glutathione (GSH) depletion was constructed to induce ferrite deposition for efficient tumor ablation (An et al., 2019). Nanozymes exhibit enhanced GSH depletion and ROS generation under photothermal conditions, leading to more efficient tumor therapeutic effects (Cao et al., 2019; Kobayashi and Choyke, 2019; Ma et al., 2019; Dong et al., 2020; An et al., 2021; Li et al., 2021; Li et al., 2023). Therefore, combining photothermal properties and photothermal therapy based on the material itself could further enhance the catalytic activity of the nanozymes, thus promoting the synergistic effect of PTT and tumor nanocatalytic therapy (Cui et al., 2013; Cui et al., 2017; Murugan et al., 2019; Yadav et al., 2019; Wang et al., 2021; Sethulekshmi et al., 2022; Feng et al., 2023; Ma et al., 2023).

Herein, we designed a photothermally enhanced m-polyethylene glycol-amine (mPEG-NH_2_)-modified vanadium-doped molybdenum disulfide nanosheets (V-MoS_2_@PEG) as a biocatalytic therapeutic platform. The hydrophilic modification of V-MoS_2_ by PEG enhances the biocompatibility of V-MoS_2_@PEG, which can be phagocytosed by the cells. After the entry of V-MoS_2_@PEG into TME, due to the specific acidic conditions, V-MoS_2_@PEG undergoes hydrolysis, and molybdenum ions (Mo^4+^) and vanadium ions (V^4+^) are released, which interact with H_2_O_2_ in TME to generate toxic ·OH, at the same time, due to the valorization of Mo ions and V ions, GSH in TME can be consumed, which results in the V-MoS_2_@PEG displaying multi-enzyme activity. Meanwhile, V-MoS_2_@PEG nanozymes exhibit a strong photothermal effect, and under 808 nm laser irradiation, a controlled temperature gradient can be generated in the local area, which warms up to 55°C within 5 min, and the multi-enzymatic activity is also dramatically increased, realizing photothermal-enhanced multi-enzymatic therapeutic effect.

## 2 Experimental section

### 2.1 Materials

Ammonium molybdate tetrahydrate ((NH_4_)_6_Mo_7_O_24_·4H_2_O, 98%), thioacetamide (C_2_H_5_NS, 98%), ammonium vanadate (NH_4_VO_3_, 95%), H_2_O_2_, dextrose monohydrate (C_6_H_12_O_6_.H_2_O, 98%), mPEG-NH_2_ (average molecular weight is 3,400), 3,3′,5,5′-tetramethylbenzidine (TMB), GSH, dimethyl sulfoxide (DMSO), and 5,5′-dithiobis(2-nitrobenzoic acid) (DNTB) were purchased from Sigma-Aldrich. RPMI 1640 medium, Methylthiazolyl tetrazolium (MTT, > 98%), fluorescein isothiocyanate (FITC, 95%), 2′,7′-dichlorofluorescein diacetate (DCFH-DA), Mouse Urea ELISA Kit, Mouse Crea (Cr) ELISA Kit, Mouse Alanine Aminotransferase (ALT) ELISA Kit, Mouse Aspartate Aminotransferase (AST) ELISA Kit, 4′,6-diamidino-2-phenylindole (DAPI), calcineurin-AM (≥97%), propidium iodide (PI, ≥ 99%), JC-1 staining kit, H&E staining, and annexin V-FITC/PI apoptosis detection kit purchased from Beyotime Biotechnology. Phosphate buffered saline (PBS) and Dulbecco’s Modified Eagle’s Medium (DMEM) were purchased from Gibco Life Technologies. All chemical reagents in this paper were not further purified.

### 2.2 Characterization

Transmission electron microscope (TEM) images showing morphology were taken with a FEI Tecnai T20 under 200 kV acceleration voltage. The crystal structure of materials was measured by X-ray diffractometer (XRD, RigakuD/MAX-TTR-III) equipped with Cu Kα radiation (λ = 0.154 nm) at 40 kV and 40 mA. The samples’ ultraviolet and visible spectrophotometric (UV-vis) absorbance spectra were acquired with a UV-1601 spectrophotometer. X-ray photoelectron spectroscopy (XPS, ESCALAB 250Xi) was applied to measure the X-ray photoelectron spectra. The surface groups were measured by Fourier Transform infrared spectrometer (FT-IR, Perkin-Elmer 580B). The generation of ROS was detected by electron spin resonance spectroscopy (ESR, Bruker D-76287). The flow cytometry assays were conducted on a BD Accuri C6 flow cytometer (United States). A confocal laser scanning microscope (CLSM, Leica TCS SP8) obtained the fluorescence image.

### 2.3 Synthesis of V-MoS_2_@PEG

(NH_4_)_6_Mo_7_O_24_·4H_2_O (1.1455 g) and C_2_H_5_NS (1.9722 g) were weighed in a 1:4 molar ratio, then a quantity of C_6_H_12_O_6_·H_2_O (0.0012 g) and NH_4_VO_3_ (0.1616 g) were added an appropriate amount of deionized water (60 mL), and stirred for 1 h until completely dissolved, after that the solution was transferred to a 100 mL polytetrafluoroethylene (PTFE) lined reactor and dried in a blast oven at 200°C for 24 h. The final product was washed three times with anhydrous ethanol and deionized water, and dried for 8 h in an oven at 60°C.

Then, 50 mg of V-MoS_2_ product was weighed in 30 mL of deionized water and 25 mg of the mPEG-NH_2_ in 20 mL of deionized water. Slowly drop the dissolved mPEG-NH_2_ into the deionized water, where the V-MoS_2_ product was dissolved and stirred at room temperature for 12 h. The product was centrifugated and dried in an oven at 60°C for 6 h to obtain the final V-MoS_2_@PEG nanozymes.

### 2.4 ROS generation estimation

To evaluate the enzymatic-like kinetics, V-MoS_2_@PEG (50 μg mL^–1^, 100 μg mL^–1^, 150 μg mL^–1^, 200 μg mL^−–1^, and 250 μg mL^–1^), TMB (10 mM), and H_2_O_2_ concentrations (final concentrations of 50 mM) were added to 2 mL of PBS solution at 25°C room temperature. The absorbance of the same material concentration was then measured after different reaction times.

### 2.5 GSH depletion estimation

V-MoS_2_@PEG (100 μg mL^–1^, 150 μg mL^–1^, 200 μg mL^−1^, and 250 μg mL^–1^) was mixed with GSH (5 mM) and H_2_O_2_ (5 mM) in PBS solution. Followed by adding DMSO at 0, 2, 4, 6, 8, and 10 min to detect–SH of GSH. The changes in absorbance at 412 nm were recorded via a UV-vis spectrophotometer.

### 2.6 Photothermal effect estimation

Different concentrations of materials (25 μg mL^−1^, 50 μg mL^−1^,100 μg mL^−1^, and 200 μg mL^−1^) were irradiated with an 808 nm laser of 0.6 W cm^−2^ power, and thermographic pictures were taken. The same concentration of V-MoS_2_@PEG solution was irradiated by 808 nm laser with different power (0.4 W cm^−2^, 0.6 W cm^−2^, 0.8 W cm^−2^, and 1.0 W cm^−2^) for 300 s at room temperature and using an infrared thermal camera (FLIR System E40) to photograph. Pure water, as the control group, was irradiated in the same way. Irradiated V-MoS_2_@PEG at 0.6 W cm^−2^ for four cycles, each composed of 300 s heating and natural cooling periods, to further test the photothermal stability.

### 2.7 *In vitro* anti-tumor estimation

The 4T1 (mouse breast cancer cells) and L929 (mouse fibroblast cells) cells were purchased from the Cell Bank. Culturing Cells in RPMI 1640 medium supplemented with 10% fetal bovine serum and 1% penicillin/streptomycin at 37°C and 5% CO_2_ humidity. The 4T1 cells were inoculated into petri dishes and cultured for 24 h, and then the medium was replaced with fresh medium containing V-MoS_2_@PEG (100 μg mL^−1^) and incubated for 0.5, 1, and 3 h, respectively. Rinsed the treated 4T1 cells twice with PBS for observation of CLSM.

Seeding the 4T1 cells into 96-well plates and culturing for 24 h. The cell culture was then renewed with a fresh medium containing different concentrations of V-MoS_2_@PEG. The light-related groups were irradiated with an 808 nm laser (0.4 W cm^−2^). 4T1 cells were handled under conditions: 1. Control; 2. NIR (808 nm, 0.4 W cm^−2^, 10 min); 3. V-MoS_2_@PEG (100 μg mL^−1^); 4. V-MoS_2_@PEG (100 μg mL^−1^) + NIR (10 min). After 4 h of co-incubation at 37°C, the NIR correlation group was subjected to 808 nm laser irradiation, followed by adding DCFH-DA (10 µM) and incubating in the dark for a further 40 min. After different treatments, the cells were rinsed twice with PBS for CLSM observation.

Seeding the 4T1 cells into 96-well plates and culturing for 24 h. The 4T1 cells were handled under conditions: 1. Control; 2. NIR (808 nm, 0.4 W cm^−2^, 10 min); 3. V-MoS_2_@PEG (100 μg mL^−1^); 4. V-MoS_2_@PEG (100 μg mL^−1^) + NIR (10 min). After 4 h of co-incubation at 37°C, the NIR correlation group was subjected to 808 nm laser irradiation, followed by a co-incubation with calcein-AM and PI for 30 min. Finally, 4T1 cells were washed repeatedly with PBS and imaged using CLSM.

Seeding the 4T1 cells into 96-well plates and culturing for 24 h 4T1 cells were handled under conditions: 1. Control; 2. NIR (808 nm, 0.4 W cm^−2^, 1 min); 3. V-MoS_2_@PEG (100 μg mL^−1^); 4. V-MoS_2_@PEG (100 μg mL^−1^) + NIR (1 min). Using Annexin V-FITC Apoptosis Detection Kit to double stain the 4T1 cells flow cytometric analysis. 4T1 cells were inoculated into petri dishes and cultured for 24 h. Then, 4T1 cells were treated with the following conditions:1. Control; 2. NIR (808 nm, 0.4 W cm^−2^, 10 min); 3. V-MoS_2_@PEG (100 μg mL^−1^); 4. V-MoS_2_@PEG (100 μg mL^−1^) + NIR (10 min). JC-1 staining of 4T1 cells was performed using the above process.

### 2.8 *In vivo* anti-tumor estimation

4-week-old female BALB/c mice were bought from Beijing Vital River Laboratory Animal Technology Co., Ltd. (Beijing, China) (1100111084356). The animal study protocol was approved by the Ethics Committee of Guangxi Medical University Cancer Hospital (protocol code KY 2022-129/130 and approved on 25 February 2022) for studies involving animals. The 4T1 breast cancer orthotopic model was established by subcutaneously injecting 1 × 10^6^ 4T1 cancer cells (100 µL) into the breast of the mouse. When tumor sizes are about ∼70 mm^3^, the tumor-bearing BALB/c mice were randomly allocated into four groups and injected with intravenous injections (*n* = 4): 1. Control; 2. NIR (808 nm, 0.4 W cm^−2^, 10 min); 3. V-MoS_2_@PEG (10 mg kg^−1^); 4. V-MoS_2_@PEG (10 mg kg^−1^) + NIR (10 min) at 0, 3, 6, and 9 d. On day 14, the 808 nm laser irradiation operations were executed. We weighed the mice and the size of the tumors every day. Tumor volume = length × width^2^/2 is the formula for calculating tumor volume. Mice were treated for a fortnight, and at the end of the treatment, mice were executed, and tumors were collected. The tumor inhibition rate of each group was calculated according to the formula of tumor inhibition rate of each group: tumor inhibition rate = (V_control_ - V_experiment_)/V_control_ × 100%. Finally, sections of tumors and major organs (heart, liver, spleen, lung, and kidney) were collected from each group for H&E staining and Ki67 assay.

### 2.9 Statistical analysis

All experimental data were used directly for statistical analyses and are expressed as mean ± S. D. The multiple comparisons among various groups were tested by the one-way analysis variance (ANOVA) with Bonferroni’s post-hoc test in the Origin 2021 software. The probability (*p*) value was presented by different numbers of asterisks (*) according to its actual value (*p < 0.05; **p < 0.01; ***p < 0.001).

## 3 Result and discussion

### 3.1 Synthesis and characterization of V-MoS_2_@PEG

**FIGURE 1:**
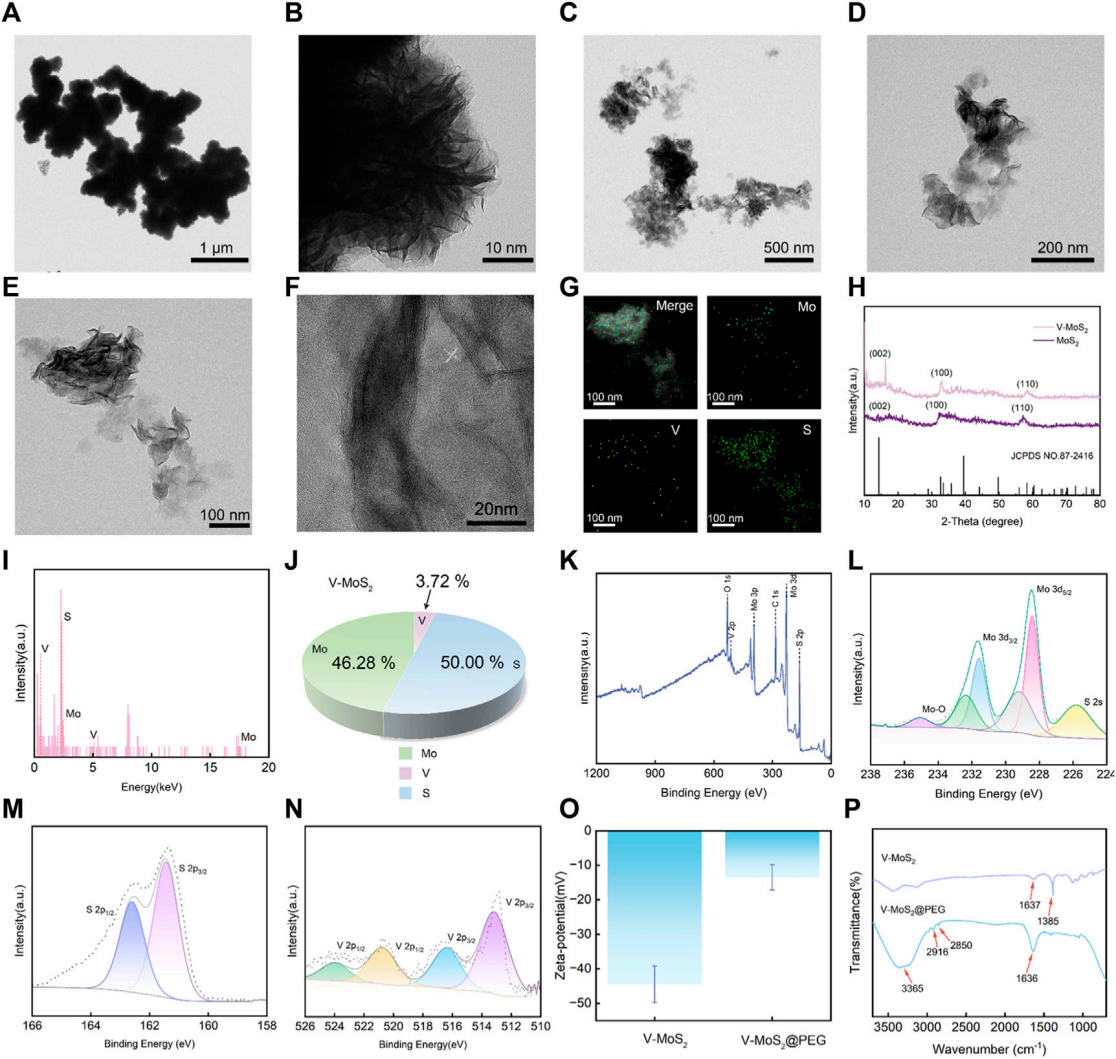
Structural characterization of V-MoS_2_ NFs. **(A,B)** TEM image of MoS_2_ NFs. **(C–E)** TEM image of V-MoS_2_ NFs. **(F)** High-resolution TEM image of V-MoS_2_ NFs. **(G)** Corresponding element mapping of the V-MoS_2_ NFs. **(H)** XRD patterns of MoS_2_ and V-MoS_2_ NFs. **(I)** EDS energy spectrum of V-MoS_2_. **(J)** V-MoS_2_ NFs elemental ratio diagram. **(K)** XPS spectra of V-MoS_2_ NFs. **(L)** Mo 3d, **(M)** S 2p, and **(N)** V 2p high-resolution XPS spectra. **(O)** Zeta-potential and **(P)** FT-IR spectra of V-MoS_2_ and V-MoS_2_@PEG NFs.

V-MoS_2_ nanoflowers (NFs) were synthesized through a two-step hydrothermal process. The MoS_2_ and the V-MoS_2_ were characterized and analyzed, and TEM images were taken for MoS_2_ and V-MoS_2_ NFs, respectively. Figures 1A, B demonstrate the nanoflowers structures formed by aggregates and single clusters of nanoflakes of MoS_2_ within the field of view, respectively, with individual MoS_2_ nanoflowers of about 50 nm and uniformly regular in size, with the edges taking on the shape of nanoflowers petals. Compared to the simple MoS_2_ NFs, the V-MoS_2_ NFs exhibit an irregular shape, while the nanoflowers become larger. As shown in Figure 1C, the V-MoS_2_ NFs could not be divided into accurate boundaries, while the shape of the nanoflowers surrounded by the lamellae became loose, and the individual V-MoS_2_ NFs still retained the nanosheet structure, which is a cluster stacked by a piece of nanosheets. As seen in Figures 1D, E, the number of the lamellae and the thickness of the lamellae are not uniform, and the lamellae size is about 150 nm approximately. These are also confirmed in the high-resolution TEM images (Figure 1F), where the lamellar structure of V-MoS_2_ NFs can be seen, with some “petals” consisting of five nanosheets and others consisting of only 2–3 nanosheets and the presence of lattice fringes is also observed, proving the existence of a lattice structure of V-MoS_2_. The ultra-small and uniform morphology create favorable conditions for the subsequent experiments. The elemental map of V-MoS_2_ NFs (Figure 1G) shows the homogeneous distributions of Mo, S, and V in V-MoS_2_ NFs, indicating the V element was successfully doped to synthesized V-MoS_2_ NFs.

In addition, the structure of V-MoS_2_ was determined by the XRD analysis (Figure 1H). The XRD patterns of MoS_2_ and V-MoS_2_ match well with the MoS_2_ monoclinic phase (JCPDS No. 87-2416). From the obtained XRD spectra, it is shown that the (002), (100), and (110) planes of MoS_2_ and V-MoS_2_ NFs are broad and pronounced, suggesting the predominance of monolayer and few-layer flower-like patterns with excellent crystallinity. After doping V ions, the characteristic V-MoS_2_ peaks showed slight shifts at 16.25°, 32.8°, and 58.4°. This may be due to the doping of the V element. The V atoms enter into the crystal structure of MoS_2_, which causes the change of the symmetry of the crystal cell and shifts the crystal faces.

The chemical composition of the V-MoS_2_ NFs was determined via energy-dispersive X-ray spectroscopy (EDS). The coexistence of Mo, S, and V signals was confirmed via the EDS analysis (Figure 1I). Hence, it can be inferred that the synthesis of V-MoS_2_ NFs was successful. Through the analysis of each elemental component in Figure 1J, it can be concluded that the Mo element accounts for 46.28%, the V element accounts for 3.72%, and the S element accounts for 50.00%, and the V doping process may be replacing some of the Mo atoms in MoS_2_. Furthermore, the near-surface chemistry of a catalyst affects its catalytic activity. XPS was used to analyze the composition of V-MoS_2_ NFs. An XPS survey spectrum showed that V-MoS_2_@PEG comprised V, C, Mo, S, and O (Figure 1K). The high-resolution XPS spectrum of Mo 3d exhibited two spin-orbit splitting, Mo 3d_5/2_ and Mo 3d_3/2_. It is shown in Figure 1L that the two highest peaks can be divided into two groups of four peaks each, indicating that V-MoS_2_ is rich in Mo^2+^. A pair of double peaks at the lower binding energy of 229.2 eV and 232.3 eV appears for Mo 3d_5/2_ and Mo 3d_3/2_ of 1T-MoS_2_, respectively. The other double high binding energy peaks belong to Mo 3d_5/2_ and Mo 3d_3/2_ of 2H-MoS_2_, respectively, suggesting that V-MoS_2_ consists of 1T and 2H phases. In particular, two small peaks were fitted at 235.1 eV and 225.7 eV, respectively. Hydrothermal synthesis may cause the oxidation of elemental Mo, with MoO_2_ (Mo^4+^), which can be seen as the affiliated peak at 235.1 eV. The slight oxidation of elemental Mo during the synthesis process changes its metallic nature to give it a lower resistivity, which may favor the occurrence of redox reactions, as this reduction in resistivity may facilitate the charge transfer of the catalyst. It can be seen that the peak intensity of V is lower, which is due to the higher density of MoS_2_ and lower doping of V, and the generated VS_2_ will be partially shielded by the shielding effect. In addition, the presence of metal sulfides (S^2-^) was analyzed by fitting the peaks to S 2p, with the two peaks for S 2p_1/2_ and S 2p_3/2_ located at 161.3 eV and 162.5 eV, respectively (Figure 1M). In the high-resolution XPS spectrum of V (Figure 1N), two peaks at 512.8 eV and 520.6 eV can be seen belonging to the V^2+^ ions of V 2p_3/2_ and V 2p_1/2_, respectively. Two other peaks, V 2p_3/2_ and V 2p_1/2_, corresponding to the V^4+^ ion, are located at 516.6 eV and 524.0 eV. XPS results show that the metal of V-MoS_2_ exists in various valence states and is relatively stable, favoring subsequent redox reactions.

Subsequently, the as-prepared V-MoS_2_ NFs were functionalized with PEG, which yielded V-MoS_2_@PEG NFs with excellent biocompatibility and physiological stability. Determination of the surface charge of V-MoS_2_ NFs used zeta potential measurements. As illustrated in Figure 1O, V-MoS_2_ NFs have tested the strength of mutual repulsion or attraction, and it can be seen that V-MoS_2_ NFs clearly show a negative potential of −44.5 mV, so the surface modification of V-MoS_2_ NFs was carried out by the addition of positively charged mPEG-NH_2_ on top of the negatively charged V-MoS_2_. The potential of V-MoS_2_@PEG was significantly lower after modification, showing a negative potential of −13.5 mV. Additionally, tests using FT-IR spectroscopy before and after modification were performed to analyze whether PEG could be successfully modified on the V-MoS_2_ NFs surface. As shown in Figure 1P, the unmodified V-MoS_2_ NFs surface has characteristic absorption peaks at 1,637 cm^−1^ and 1,385 cm^−1^ due to the uncleaned carboxyl groups left on the surface during the preparation process. The modified V-MoS_2_ NFs have prominent characteristic absorption peaks (3,365 cm^−1^, 2,916 cm^−1^, 2,850 cm^−1^, 1,636 cm^−1^, and 3,365 cm^−1^) of NH_2_, -CH_3_, -CH_2_, and C-O-C groups associated with mPEG-NH_2_, indicating that mPEG-NH_2_ was successfully modified onto the surface of V-MoS_2_ NFs. As a result, the negatively charged V-MoS_2_ NFs were deposited on the positively charged mPEG-NH_2_ by decisive electrostatic action to obtain the final V-MoS_2_@PEG composites.

### 3.2 *In vitro* catalytic activity of V-MoS_2_@PEG

**FIGURE 2:**
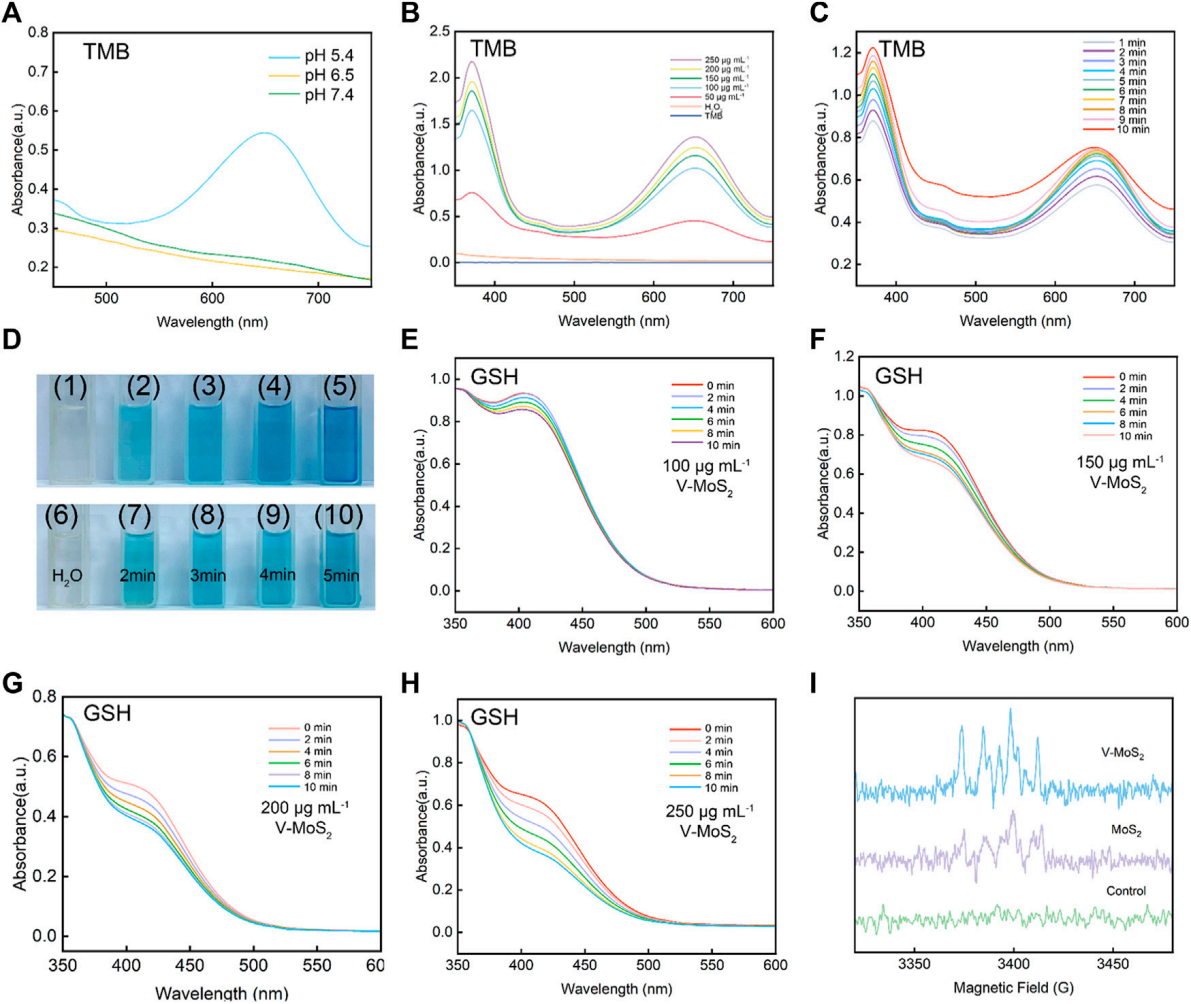
ROS production capacity of V-MoS_2_ NFs. **(A)** Oxidation of TMB to produce ·OH in different pH environments (V-MoS_2_ 200 μg mL^−1^). **(B)** Material concentration-dependent oxidation of TMB indicates ·OH generation under different conditions. **(C)** Time-dependent oxidation of TMB indicates ·OH generation under different conditions. **(D)** Color response of TMB after being oxidized, (1) and (6) are controls, (2)–(5) correspond to the color response of TMB after 5 min of reaction with 50, 100, 150, and 200 μg mL^−1^, respectively, and (7)–(10) correspond to the picture after 2–5 min of reaction with 200 μg mL^−1^, respectively. **(E–H)** Absorbance spectra of glutathione depletion over 0–10 min of different substance concentrations (GSH 5 mg mL^−1^). **(I)** ESR spectra for V-MoS_2_ NFs and MoS_2_ NFs in the presence of DMPO and H_2_O_2_ (V-MoS_2_ 200 μg mL^−1^; MoS_2_ 200 μg mL^−1^; H_2_O_2_ 0.5 mM).

The abundant Mo^2+^ and the photothermal triggering property produced a large amount of ·OH, which was confirmed in subsequent experiments. After mixing V-MoS_2_@PEG with H_2_O_2_ solution, the production of ·OH was detected by varying the solution acid-base values (pH = 5.4, 6.5, and 7.4) (Figure 2A). The ·OH production could be detected at pH = 5.4, whereas slight ·OH production signals were detected at pH 6.5 and 7.4, suggesting that V-MoS_2_ has better pH responsiveness and can hydrolyze ·OH using H_2_O_2_ from TME in the acidic environment of TME. The solution pH was controlled to 5.4, and the H_2_O_2_ concentration was unchanged. The H_2_O_2_ decomposed into ·OH (Figure 2B) in a material-concentration (50, 100, 150, 200, and 250 μg mL^−1^)-dependent manner, controlling the material concentration is constant (150 μg mL^−1^) H_2_O_2_ decomposes to ·OH in a time-dependent manner, and these can be detected by the TMB substrate (Figure 2C). As the concentration of the material increases, more ·OH is generated, while the generated ·OH can effectively accumulate over time. Figure 2D shows photographs of TMB color development at different material concentrations and times for the same concentration, respectively. The change in the color of TMB becomes darker and darker with the concentration of the material and the time it is left in place, which is consistent with the results of the UV test of TMB. As shown in Figures 2E–H, to verify the effective depletion of glutathione by V-MoS_2_, 5 mg mL^−1^ of GSH was added using different material concentrations (100, 150, 200, and 250 μg mL^−1^), and the glutathione absorbance at different times was detected after different times of reaction. A concentration-dependent and time-dependent trend in glutathione depletion can be found; the decrease in UV absorption peaks indicated the degradation of GSH, suggesting that V-MoS_2_ could circulate in TME with sufficient GSH and H_2_O_2_ and generate ·OH to kill tumor cells. In addition, an ESR assay using DMPO was performed to detect the production of ·OH under different conditions. As shown in Figure 2I, MoS_2_ and V-MoS_2_ solutions were mixed in H_2_O_2_, respectively, and a clear signal could be observed, whereas there was no signal strength was detected in the control group, which proved that V-MoS_2_ has a more desirable ·OH generation ability compared to MoS_2_, Further evidence that the doping of element V enhances the generation of ·OH.

### 3.3 Characterization of the photothermal performance of V-MoS_2_@PEG

**FIGURE 3:**
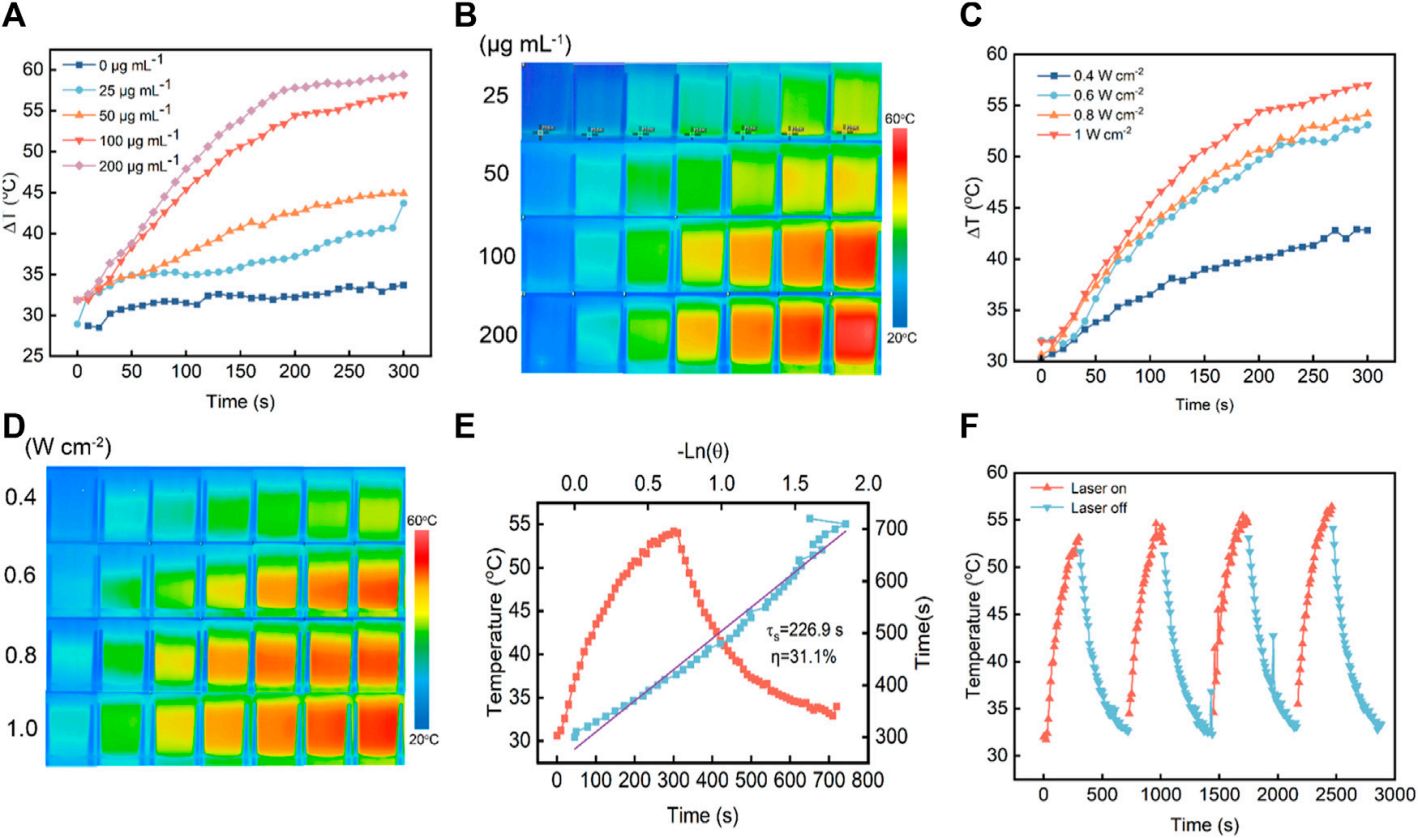
Inspection of photothermal performance of V-MoS_2_@PEG NFs. **(A)** Warming curves of different concentrations of materials under 808 nm NIR irradiation within 5 min. **(B)** Infrared thermograms of different concentrations of materials under 808 nm NIR irradiation (0–3.5 min, photographed at 30 s intervals). **(C)** Warming curves of materials under 808 nm NIR irradiation at different power intensities within 5 min (V-MoS_2_@PEG 100 μg mL^−1^). **(D)** Infrared thermograms of the materials under 808 nm NIR light irradiation at different power intensities (0–3.5 min, photographed at 30 s intervals) **(E)** Photothermal stability of V-MoS_2_@PEG NFs under 808 nm laser (0.8 W cm^−2^) for 5 min and cooling. **(F)** Photothermal stability of 100 μg mL^−1^ V-MoS_2_@PEG NFs and 0.6 W cm^−2^ under 808 nm laser irradiation for four on/off cycles.

Inspired by these excellent properties of V-MoS_2_@PEG NFs, *in vitro* photothermal-enhanced nanocatalytic therapeutic efficacy was evaluated with 808 nm near-infrared (NIR) light. The photothermal effect of V-MoS_2_@PEG NFs was investigated by irradiating different concentrations of V-MoS_2_@PEG NFs solution using an 808 nm laser. In Figure 3A, the temperature change curves of the V-MoS_2_@PEG NFs with different concentrations were measured during the 5 min heating process, and it can be seen that compared with the temperature change curves of pure water, V-MoS_2_@PEG NFs have better photothermal effect, and the temperature change curves become more and more obvious as the concentration continues to rise, while the temperature of pure water changed slightly. In Figure 3B, this temperature change is reflected by a thermal infrared map. Different concentrations of the V-MoS_2_@PEG were set up and given 808 nm laser irradiation for 3.5 min respectively, and photographs of the infrared thermograms were taken at intervals of 30 s, and a clear temperature change of the V-MoS_2_@PEG NFs could be observed. In Figure 3C, the concentration of V-MoS_2_@PEG NFs was set to 100 μg mL^−1^ and measured the temperature rise curve over time at different powers of the 808 nm laser (0.4 W cm^−2^, 0.6 W cm^−2^, 0.8 W cm^−2^, and 1.0 W cm^−2^). With the increase of power, the temperature rise rate of V-MoS_2_@PEG significantly increases, with power and concentration-dependent photothermal properties. In Figure 3D, these photothermal changes were reflected in an infrared thermal image; it can be observed that as the 808 nm laser power increases, the temperature change of V-MoS_2_@PEG becomes more and more obvious, with a good photothermal effect. In addition, with the 0.8 W cm^−2^ 808 nm laser irradiating, the curve can be fitted according to the heating-cooling curve, and the heat transfer time constant of the system is obtained as 226.9 s. V-MoS_2_@PEG NFs have a great photothermal conversion efficiency as 31.1% (Figure 3E). The great photostability of V-MoS_2_@PEG NFs was also verified by three laser power on/off cycles, in which the photothermal effect showed only a slight change (Figure 3F), which indicated that V-MoS_2_@PEG has excellent photothermal stability.

### 3.4 *In vitro* cytotoxicity of V-MoS_2_@PEG

**FIGURE 4:**
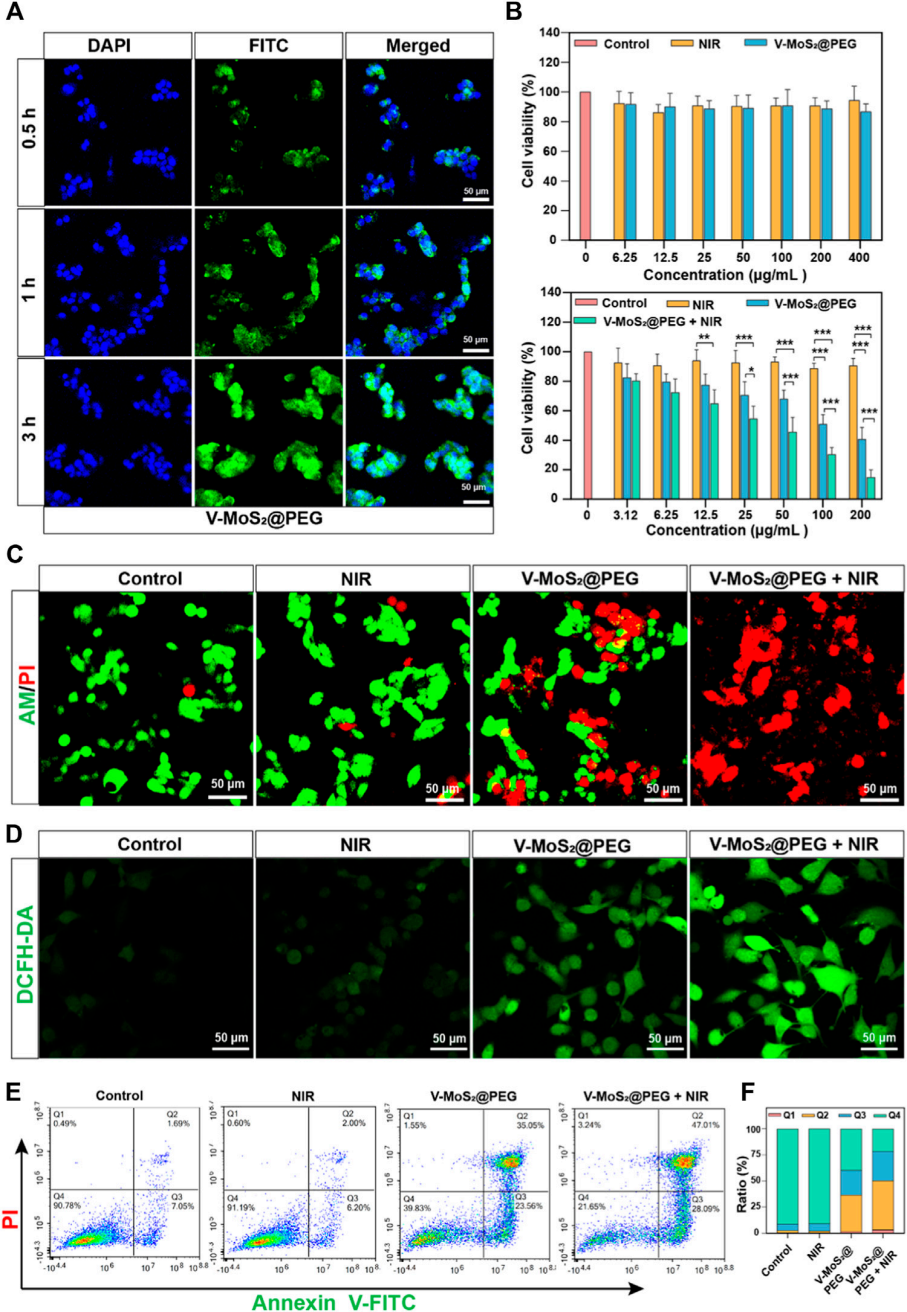
Inspection of photothermal performance. **(A)** CLSM images and corresponding line-scan profiles of 4T1 cells incubated with V-MoS_2_@PEG NFs (100 μg mL^−1^) at different time intervals. **(B)** Viability of L929 cells and 4T1 cells cultured with different concentrations of V-MoS_2_@PEG (the cell viability of 4T1 cells cultured with different concentrations and given 808 nm NIR light stimulation of V-MoS_2_@PEG). **(C)** Calcein-AM/PI double staining: 1) control, 2) 808 nm laser, 3) V-MoS_2_@PEG, 4) V-MoS_2_@PEG + 808 nm laser. **(D)** CLSM images and **(D)** the fluorescence quantification of 4T1 cells stained with DCFH-DA after different treatments. **(E)** Fluorescein-annexin V and PI staining assay, and **(F)** the corresponding quantitative analysis of 4T1 cells handled with different formulations. The data are presented as mean ± standard deviation (S.D.) (*n* = 3) with *p < 0.05, **p < 0.01, ***p < 0.001.

To evaluate the cellular internalization performance of V-MoS_2_@PEG NFs, we labeled it with FITC and denoted it as FITC-labeled V-MoS_2_@PEG NFs. At different time points, the *in vitro* cellular take-up of FITC-labelled V-MoS_2_@PEG by 4T1 cells was evaluated using CLSM. As given in Figure 4A, the fluorescence signals of FITC became stronger with a prolonged incubation time, exhibiting that the V-MoS_2_@PEG exhibited great cellular uptake efficiency. As mentioned above, tumor cells are more susceptible to electrical charges than normal cells, thus allowing more drugs to enter the tumor cells, enabling passive targeting functions and accumulation of more drugs at the tumor site. The biocompatibility and therapeutic efficacy of normal cells and tumor cells were evaluated using an MTT assay (Figure 4B). Repeated the experiment three times (*n* = 3). There were no significant changes in L929 cell survival compared to the control when we only gave the NIR irradiation or different concentrations of V-MoS_2_@PEG NFs. However, the 4T1 cells were handled with different treatments. At the V-MoS_2_ and the V-MoS_2_@PEG + NIR groups, concentration-dependent cytotoxicity was exhibited in 4T1 cells with a significant reduction in cytotoxicity, indicating that V-MoS_2_@PEG had a photothermally-enhanced effect of promoting cells death, which due to the collaborative effect of nano-enzymes and pyroelectric catalysis. After treatments with different experimental groups, the cells were co-stained with calcein-AM and PI to visually distinguish the living cells from dead cells. As shown in Figure 4C the control groups and 808 nm laser irradiation group had almost no cell damage, while a majority of the dead cells were observed in the V-MoS_2_@PEG plus 808 nm laser and the V-MoS_2_@PEG groups, indicating efficient photothermal and ·OH-enabled catalytic therapeutic performances, respectively. Measurement of the degree of intracellular oxidative stress and analysis of ·OH production after co-culture with different samples. In Figure 4D, the ·OH was determined using DCFH-DA as a fluorescent probe. Compared with the other three groups, the ·OH level was significantly increased in V-MoS_2_@PEG + NIR group, which indicated that NIR combined with V-MoS_2_@PEG could achieve higher ·OH production. Generally, ·OH production would increase intracellular oxidative stress, promoting cell death. The result of the flow cytometer displayed a similar trend (Figure 4E). The control group or NIR group did not cause cell apoptosis. The V-MoS_2_@PEG and the V-MoS_2_@PEGNIR groups showed partial apoptosis due to the enzyme-like activity of the V-MoS_2_@PEG and the efficient photothermal of V-MoS_2_@PEG. Due to the V-MoS_2_@PEG has better photothermal properties and ·OH-producing enzyme-like activity, under 808 nm laser irradiation, the vibration between the internal lattices of V-MoS_2_@PEG was intensified, and the V ions and Mo ions became more active, which enhanced the intracellular redox reaction, increased the ·OH-producing efficiency, at the same time, laser irradiation also results in cell hyperthermia, which promotes the process of apoptosis. Figure 4F showed that the apoptotic ratio in the V-MoS_2_@PEG + NIR group reached 75.10% (sum of Q2 + Q3), which was significantly higher than those of the control group (8.74%), NIR group (8.20%), and V-MoS_2_@PEG group (58.61%). As expected, the 4T1 cells treated with V-MoS_2_@PEG + NIR showed the most apoptosis, providing strong evidence that photothermal-induced pyroelectric catalysis and intrinsic nanozymes catalysis co-enhanced ·OH generation, ultimately leading to apoptosis of a significant number of tumor cells. These results show the high synergistic therapeutic efficacy of V-MoS_2_@PEG enabled photothermal-enhanced tumor nanocatalytic therapy, in which huge amounts of highly toxic ·OH and local hyperthermia could be produced to induce apoptosis in the 4T1 cells.

### 3.5 *In vivo* anticancer effect of V-MoS_2_@PEG

**FIGURE 5:**
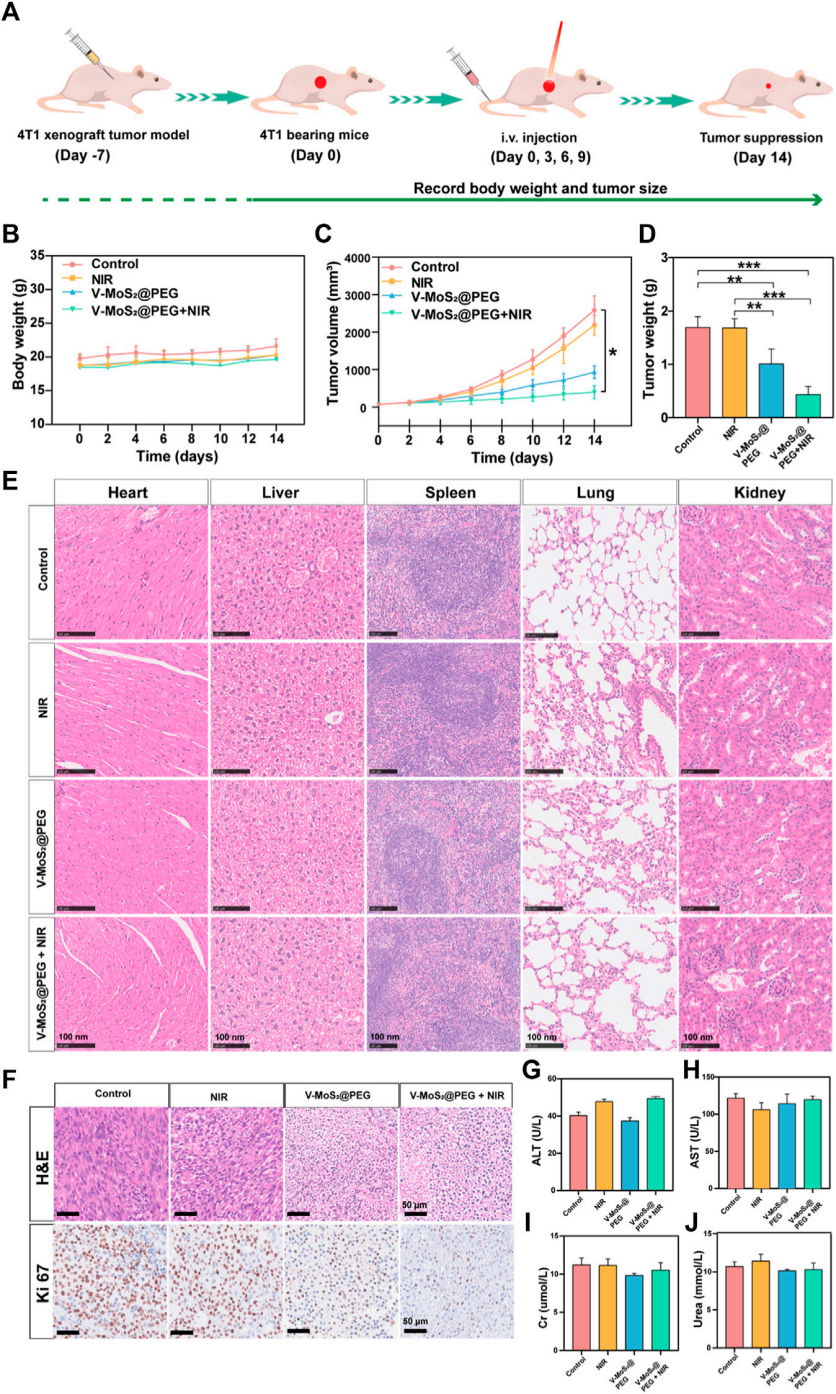
*In vivo* treatment of mice with 4T1 tumors. **(A)** Schematic representation of the establishment of the 4T1 tumor model. **(B)** Mouse weights and **(C)** tumor volumes in different groups during the 14-day treatment. **(D)** Tumors weights in various groups: 1) control, 2) NIR, 3) V-MoS_2_@PEG, 4) V-MoS_2_@PEG + NIR (*n* = 4 biologically independent mice, *p < 0.05, **p < 0.01, ***p < 0.001, ns indicating no significant differences) **(E)** Tissue section images of heart, liver, spleen, lung, and kidney in different groups. **(F)** H&E of tumors after different treatments and Ki67 staining assay. **(G–J)** Detection of liver and kidney functions in different treatment groups.

We further studied the *in vivo* tumor suppressive efficiency of V-MoS_2_@PEG in 4T1 tumor-bearing mice. The BALB/c mice were randomly allocated into four groups and injected with intravenous injections (*n* = 4): 1. Control; 2. NIR (808 nm, 0.6 W cm^−2^, 10 min); 3. V-MoS_2_@PEG (10 mg kg^−1^); 4. V-MoS_2_@PEG (10 mg kg^−1^) + NIR (10 min) at 0, 3, 6, and 9 d. On day 14, 808 nm laser irradiation operations were executed (Figure 5A). Subsequently, we investigated its suppressed effect in tumor-bearing mice. After treatment completion, the subcutaneous tumors were collected and weighed. Every 2 days, the weights and subcutaneous tumor volumes of the mice were measured (Figures 5B, C), and during the entire treatment process, the changes in mice weight in each group were similar. The mice exhibited negligible weight loss throughout the entire treatment, proving the limited adverse effects of the treatments (Figure 5B). The tumor growth curves (Figure 5C) showed that the relative tumor volumes of the NIR group were comparable with those of the control group, suggesting that pure NIR irradiation could slightly inhibit tumor growth. The V-MoS_2_@PEG group inhibited tumor growth, confirming that its enzyme activity could kill the tumors, but the effect was limited. The V-MoS_2_@PEG + NIR group exhibited the highest tumor inhibitory effect, and the tumor was almost completely inhibited, owing to the NIR-mediated enzymatic and pyroelectric catalysis. Tumor weight changes were quantified for each group, as shown in Figure 5D, where it can be seen that the most significant changes were seen in the V-MoS_2_@PEG + NIR group. At the end of the treatment, H&E staining images of normal organs after treatment had no apparent tissue damage (Figure 5E), suggesting that V-MoS_2_@PEG possessed superior *in vivo* biocompatibility. H&E staining and Ki67 staining of tumor sections from each group were conducted to demonstrate the excellent tumor suppression effects, indicating that the tumor tissues in the V-MoS_2_@PEG group were severely damaged, while the tissues in the control group and NIR groups exhibited no significant changes (Figure 5F). Similarly, the damage was most severe after the V-MoS_2_@PEG + NIR treatment, and the ALT, AST, Cr, and Urea were used to assess the liver and kidney functions. As shown in Figures 5G–J, no significant damage was observed in the major organs during treatment. These results confirm that V-MoS_2_@PEG promotes apoptosis *in vivo* and can be applied as an innovative nanoplatform for cancer therapy.

## 4 Conclusion

In summary, we developed a photothermal therapy/enzyme-catalyzed anti-tumor platform (V-MoS_2_@PEG NFs), which was synthesized using a hydrothermal reaction. The *in vitro* and *in vivo* experiments showed that the Fenton-like reaction based on the redox of Mo and V ions in the TME could achieve the conversion of H_2_O_2_ into ·OH while effectively consuming GSH for efficient nanocatalytic tumor therapy in the NIR window (808 nm laser irradiation), and the transfer of electrons during the redox process could also achieve the continuous supply of enzyme-like activity. This was achieved by coating V-MoS_2_ NFs with PEG into the V-MoS_2_@PEG nanocomposites. The charge attraction between PEG and V-MoS_2_ has led to a significant increase in the V-MoS_2_@PEG stability, improving the material’s biocompatibility. The V-MoS_2_@PEG nanozymes acted as a highly efficient Fenton-like agent to generate highly toxic ·OH in specific acidic tumor microenvironments. In addition, the V-MoS_2_@PEG nanozymes exhibited GSH-consumption capability, which impaired the antioxidant defenses of tumor cells and made the cells more sensitive to ROS, thereby further improving the ·OH-mediated tumor nanocatalytic therapy. Most importantly, V-MoS_2_@PEG-enabled photothermal hyperthermia in the NIR-II region significantly enhanced the ability to produce toxic ·OH and the depletion of GSH. As a result, the V-MoS_2_@PEG exerts both enzyme-like activity and photothermal therapy, resulting in enhanced membrane permeability of tumor cells, which improves the chances of nanomedicine deposition and produces an excellent therapeutic effect.

## Data Availability

The original contributions presented in the study are included in the article/Supplementary Material, further inquiries can be directed to the corresponding authors.
